# A biomimetic approach towards a universal slippery liquid infused surface coating

**DOI:** 10.3762/bjnano.15.111

**Published:** 2024-11-08

**Authors:** Ryan A Faase, Madeleine H Hummel, AnneMarie V Hasbrook, Andrew P Carpenter, Joe E Baio

**Affiliations:** 1 School of Chemical Biological and Environmental Engineering, Oregon State University, Corvallis, OR 97331, USA

**Keywords:** biocompatibility, biomimetic, blood-contacting, hemocompatibility, non-fouling

## Abstract

One biomimetic approach to surface passivation involves a series of surface coatings based on the slick surfaces of carnivorous pitcher plants (Nepenthes), termed slippery liquid-infused porous surfaces (SLIPS). This study introduces a simplified method to produce SLIPS using a polydopamine (PDA) anchor layer, inspired by mussel adhesion. SLIPS layers were formed on cyclic olefin copolymer, silicon, and stainless steel substrates, by first growing a PDA film on each substrate. This was followed by a hydrophobic liquid anchor layer created by functionalizing the PDA film with a fluorinated thiol. Finally, perfluorodecalin was applied to the surface immediately prior to use. These biomimetic surface functionalization steps were confirmed by several complimentary surface analysis techniques. The wettability of each surface was probed with water contact angle measurements, while the chemical composition of the layer was determined by X-ray photoelectron spectroscopy. Finally, ordering of specific chemical groups within our PDA SLIPS layer was determined via sum frequency generation spectroscopy. The hemocompatibility of our new PDA-based SLIPS coating was then evaluated by tracking FXII activation, fibrin generation time, clot morphology, and platelet adhesion to the surface. This hemocompatibility work suggests that PDA SLIPS coatings slow or prevent clotting, but the observation of both FXII activation and the presence of adherent and activated platelets at the PDA SLIPS samples imply that this formulation of a SLIPS coating is not completely omniphobic.

## Introduction

Clot formation and the overall compatibility of artificial materials within the body remains a common complication of blood contacting surfaces [1–8]. A critical hurdle in reducing the thrombogenicity of a material lies in addressing the intricate layer of protein within blood that adsorbs to any surface it comes into contact with. This layer is responsible for the initiation of host responses such as coagulation or inflammation. To address the body’s reaction to these materials, researchers have developed strategies to combat blood clots and bacterial infections in frequently used devices such as tubing, catheters, and grafts [9–15]. Consideration of the coagulation cascade is a crucial part in engineering new materials that interact with blood and reducing the potential for adverse effects. Factor XII, thrombin, and calcium are critical components of the coagulation cascade, and their removal represents a pathway for lowering thrombus formation due to contact with foreign materials. Each of these components has led to different approaches for the removal and or repulsion of these important molecules that control the clotting cascade. The most prominent of the methods are ones that aim to reduce non-specific protein adsorption, increase adhesion resistance, use biomolecules to remove targets of interest, and enhance endothelial cell attachment.

One biomimetic approach to surface passivation involves a series of surface coatings based on the slick surfaces of carnivorous pitcher plants (Nepenthes), termed slippery liquid-infused porous surfaces (SLIPS) [4]. When wetted, the slippery surfaces on the plant cause prey to slide into the bottom of the pitcher-like feature, where they are digested by the plant. Like the pitcher plant surfaces, SLIPS can repel adhesion through the formation of a liquid–liquid interface, unlike more standard surface passivation techniques that consist of a solid–liquid interface [16].

Within a SLIPS coating, a lubricant is anchored to a substrate, creating a smooth liquid layer that is energetically favored to interact with the solid surface. The surface chemistry of the anchoring layer is crucial to the viability of the overall coating as the lubricant must have a greater affinity for the substrate surface than for any foreign surface. Materials coated with SLIPS have demonstrated effectiveness in resisting corrosion, reducing bio-fouling, and preventing icing [1–3,6,8]. There is also evidence that SLIPS are a promising strategy for increasing the biocompatibility of materials [4,11].

SLIPS are generally defined by either a porous or flat solid surface that consists of chemistry that is similar to the pervading liquid [11,17]. The substrate serves an anchor to a lubricant, which provides a smooth liquid layer that provides some sort of resistance to surface adhesion. The liquid is anchored to the surface through van der Waals forces, and capillary forces if there is a rough surface, forces which give way to conditions that are energetically favorable to the retention of the infused liquid as opposed to a foreign one. The preparation of SLIPS substrates include plasma treatments [4,12], acid/base soaks [1,13], anodization [3,14], silane chemistry [18], and polymer multilayers [5,15]. While these methods are effective, there is often a requirement for a specific chemical environment, like a plasma processing step or the growth of an oxide layer. In this investigation, we aim to simplify the fabrication of SLIPS by producing the required lubricant anchor layer through the polymerization of dopamine. This dopamine polymerization step is extremely simple and will form a “sticky” layer to almost any chemistry, thereby, providing a straightforward process to produce a SLIPS layer on almost any substrate.

**Figure 1:**
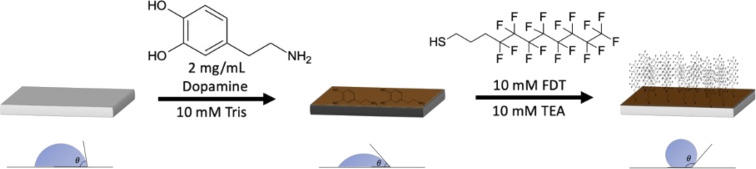
Schematic of PDA–FDT–PFD coating process. The bare substrate, cyclic olefin copolymer (COC, left) is hydrophobic. A layer of polydopamine (PDA) is added to the surface by incubating surfaces overnight with dopamine and Tris. PDA is hydrophilic, so the PDA coating step can be verified through water contact angle measurements. Fluorinated dodecanethiol (FDT) was conjugated to the PDA surface by incubating overnight. FDT is hydrophobic, so this step can be also verified through water contact angle measurements. Not pictured is the addition of perfluorodecalin (PFD) to the surface immediately prior to use.

Over a decade ago, Lee et al. demonstrated that mussels could adhere to virtually any surface through a molecule called polydopamine (PDA) [19]. The power of PDA lies in its ability to coat a thin layer onto any material, from polymers and metals to glass. The authors also demonstrated the modification of the PDA film though the formation of a pseudo self-assembled monolayer [19–20]. In this process, molecules that contain thiol groups attach to the newly formed PDA surface via Michael addition [21–23]. In this work, we take advantage of PDA to covalently attach fluorinated chains, producing the foundation of a SLIPS material (Figure 1).

In this work we propose and characterize a two-step process, forming a SLIPS layer via a sticky PDA film, to modify the surface of three different materials. To test this combination of PDA and SLIPS we used three distinct materials, cylic olefin copolymer (COC), silicon, and 316 stainless steel (SS) as our substrates. These substrates were first coated with PDA; then, a fluorinated thiol was attached to serve as the anchor for the infused fluid. The resulting surface modifications were then characterized by water contact angle measurements, atomic force microscopy (AFM), sum frequency generation spectroscopy (SFG), and X-ray photoelectron spectroscopy (XPS). Measuring static water contact angles is a straightforward method to determine the relative wettability of a material and allows for a quick check if our surface modifications were successful with a fluid similar to blood [24]. AFM is a technique that provides topographical information through a nanoscale probe [25]. After each successive layer of the coating the topography of the surface will change and can be measured via AFM. SFG is a surface-sensitive non-linear spectroscopic technique, which has the ability to probe vibrational modes at an interface, thereby, providing insight into the order and confirmation of molecules at an interface [26–27]. XPS is a surface-sensitive technique to determine the atomic composition of the outer ca. 10 nm of a surface [28].

As mentioned above, SLIPS represent a novel method of surface passivation that involves trapping a liquid, often an oil, onto a porous surface [4,29]. The result is an antifouling surface that has been proposed to resist adhesion of coagulation factors [4,29]. Yet, the activation of coagulation factors, specifically FXII, and the plasma clotting kinetics on SLIPS surfaces have not been studied previously. Therefore, to assess the hemocompatibity of our newly created PDA-based SLIPS coating, we tested the resistance of the coating to FXII activation, clot formation, clot stability, and platelet adhesion.

FXII activation gives insight into the extent of intrinsic coagulation. On biocompatible surfaces, we would expect to see a lower concentration of activated FXII (FXIIa) than on prothrombogenic surfaces. Platelet adhesion and activation testing describes the tendency of platelets to adhere to a surface, which promotes coagulation. We would expect to see fewer adherent platelets on biocompatible surfaces compared to prothrombogenic surfaces. Fibrin generation time describes the resistance to fibrin formation from fibrinogen and, thus, clot formation. Biocompatible coatings will have a longer fibrin generation time than prothrombogenic coatings. Clot stability, quantified via crosslinking density measurements, provides insight into the ability of a clot to break down. Clots on biocompatible coatings should be less stable (larger fiber diameter and lower crosslinking density) compared to prothrombogenic surfaces.

These assays collectively provide a starting point for understanding the foundation for understanding the behavior of PDA SLIPS when in contact with blood. We anticipate that the PDA SLIPS coating will demonstrate antithrombogenic properties. Therefore, we expect it to exhibit a lower concentration of FXII, decreased platelet adhesion, longer fibrin generation time, and lower clot stability compared to other surfaces.

## Experimental

### Sample preparation

COC (microfluidic ChipShop, Jena, Germany) was first cut to size, into 1 × 1 cm^2^ squares. These COC substrates were cleaned by sequential sonication in DI water and then in a 1:1 mixture of ethanol and acetone for 30 min. Si substrates were fabricated via a diamond-tipped blade in a Disco wafer saw (Disco, Tokyo, Japan) from a silicon wafer cut into 1 × 1 cm^2^ substrates. These Si substrates were left overnight to soak in DI water and then sonicated in 20 min intervals with dichloromethane, acetone, and ethanol. 316 SS was purchased from McMaster-Carr (Elmhurst, IL) and ground up to grit 3000 with SiC emery paper and cut by hand into 1 × 1 cm^2^ chips. The cut SS substrates were rinsed in DI water and ethanol followed by sonication in a 1:1 mixture of chloroform and methanol for 20 min [30]. All substrates were then dried under a stream of nitrogen and sealed until use. The cleaned substrates were then immersed in dopamine hydrochloride (Sigma-Aldrich, St. Louis, MO) at a concentration of 2 mg/mL for 24 h in a 10 mM Trizma base (Sigma-Aldrich, St. Louis, MO) solution at a pH of 8.5. PDA-coated samples were rinsed with DI and then introduced to a 10 mM aqueous solution of 1*H*,1*H*,2*H*,2*H*-perfluorodecanethiol (FDT) (Sigma-Aldrich, St. Louis, MO) and triethylamine [19]. Following FDT functionalization, the samples were thoroughly rinsed and sonicated in DI water and then sealed under nitrogen until ready to use*.* Immediately prior to use, the SLIPS formation was completed by adding a layer of liquid perfluorodecalin (PFD) to the surface (Figure 1).

### Water contact angle

A custom setup was used similar to one previously described [31]. Static contact angle measurements were conducted with the sessile drop method. Droplets of 5 µL were pipetted onto the surface, and an image was captured. Eight images from two duplicates of each sample type were acquired on a smartphone device and processed in ImageJ (NIH).

### Atomic force microscopy

AFM was conducted on a Veeco di Innova instrument in tapping mode. RTESPA-300 (Bruker, Billerica, MA) probes were used with a tip radius of 12 nm, a spring constant of 40 N/m, and a frequency of 300 kHz. A minimum of six images from two different samples were produced with dimensions of 5 × 5 μm at a scan rate of 0.5 Hz. Post processing and analysis of the collected scans included a lowpass filter and took place in the “NanoScope analysis” software.

### Sum frequency generation vibrational spectroscopy

The SFG setup used an EKSLPA Nd:YAG laser operated at 50 Hz to generate a fixed visible (532 nm^−1^) and tunable infrared beam (1000–4000 cm^−1^). Each beam was overlapped in space and time to produce SFG photons at a spot size of approximately 1 mm. Spectra were collected and offset with a 4 cm^−1^ step size at 200 acquisitions per step. Spectra were collected in an ssp, ppp, and sps polarization combinations (SFG, visible, IR) in a wavenumber range of 1100–1800 cm^−1^. The spectra were then normalized by dividing the signal by the visible and infrared intensities.

### X-ray photoelectron spectroscopy

Spectra were collected on a PHI 5600 system with a hemispherical analyzer and a monochromatic Al Kα (1486.6 eV) X-ray source with an electron flood gun. Scans were collected with a takeoff angle of 55° at a pressure below 3 × 10^−9^ Torr. A pass energy of 187.5 eV with a step size of 0.8 eV was used for the survey scans, and the high resolution had a pass energy of 23.5 eV and a step size of 0.5 eV/step. Spectra were collected with an X-ray spot size of 400 μm at three different spots for each sample. Analysis and peak fitting took place with CasaXPS (Casa Software Ltd.), where a linear background was used.

### Statistical analysis

All statistical analysis was performed using JMP (SAS Institute Inc., Cary, NC). For each test, a one-way ANOVA and a Tukey post-hoc comparison were performed to determine differences between means. For biocompatibility data that was normalized to glass, the error from glass is imbedded in the reported standard deviation. Sample sizes for presented data is included in the figure captions and reported as the mean ± (standard deviation).

### Preparation of platelet-poor plasma and washed platelets

Human blood samples were collected from volunteers at Oregon State University Student Health Services in accordance with an approved Institutional Review Board (IRB-2019-0271). Informed consent from four different healthy human donors was obtained prior to venipuncture. Volunteers were above 110 lbs, over the age of 18 years, and had no medication use two weeks prior to the blood draw. Blood samples were processed following the method of McCarty and colleagues [32]. Approximately 15 mL of blood was drawn into sodium citrate Vacutainers. Acid-citrate-dextrose (ACD) was added to the whole blood at a 1:10 volume ratio. Blood was centrifuged at 200*g* for 20 min at room temperature. The supernatant platelet-rich plasma was transferred, and 0.1 μg/mL prostaglandin I2 was added to inhibit platelet activation. Platelet-rich plasma was centrifuged at 1000*g* for 10 min at room temperature. The supernatant platelet-poor plasma (PPP) was transferred and used for fibrin generation studies. The platelet pellet was resuspended in Tyrode’s buffer (129 mM NaCl, 20 mM HEPES, 12 mM NaHCO_3_, 2.9 mM KCl, 1 mM MgCl_2_, and 0.34 mM Na_2_HPO_4_), and 0.1 μg/mL prostaglandin I2 was added. The platelets were centrifuged at 1000*g* for 10 min, the supernatant was discarded, and pelleted platelets were resuspended in Tyrode’s buffer. Platelets were counted using a hemocytometer and the platelet solution was diluted to a concentration of 1 × 10^8^ platelets/mL [33–34].

### FXIIa assay

Wells of interest of a nontreated 96-well polystyrene were blocked with 300 μL 1% bovine serum albumin (BSA) in Milli-Q water for 2 h at 37 °C. After 2 h, the wells were rinsed three times with Milli-Q water. Plates were coated immediately prior to use. FXIIa assays were performed using methods adapted from Bates and colleagues [33]. A solution of FXII (200 nM), prekallikrein (PK) (50 nM), and high-molecular-weight kininogen (HMWK) (50 nM) in vacuum-filtered and degassed buffer (25 mM HEPES pH 7.4, 150 mM NaCl, and 0.1% BSA) was prepared, and 120 μL was added to each well of a 96-well plate blocked with BSA. After incubation for 60 min, 90 μL of protein solution was removed and added to a new plate containing 5 μL 4.7mM soybean trypsin inhibitor (Sigma). 5 μL of chromogenic substrate S-2302 (Chromogenix) was added to each well and the absorbance was measured at 405 nm every 1 min for 60 min using a FlexStation 3 microplate reader (Molecular Devices). A standard curve was used to determine the concentration of FXIIa from the measured OD. A BSA-blocked well was used as a negative control and glass was used as a positive control. Three separate experiments were performed for a total sample size of 18.

### Clot turbidity analysis

Wells of interest of a non-treated polystyrene 96-well plate were blocked with 300 μL 1% BSA in Milli-Q water for 2 h at 37 °C. After 2 h, wells were rinsed three times with Milli-Q water. Plates were coated immediately prior to use. Fibrin generation experiments were performed on a FlexStation 3 (Molecular Devices) using methods adapted from Bates et al. and Sask et al. [33,35]. 50 μL of PPP from fresh human blood samples from two donors was added to wells with or without surfaces, along with 50 μL buffer (25 mM HEPES pH 7.4, 150 mM NaCl). Plasma was then recalcified with 50 μL 25 mM CaCl_2_. Absorbance measurements were taken every 1 min for 60 min at 405 nm. Three separate experiments were performed for a total sample size of 18 per donor. Fibrin generation time was defined as the time to reach a 5% increase over the baseline absorbance value. 5% is a sufficient threshold to overcome the noise floor of the data [33].

### Clot morphology

Wells of interest of a non-treated 96-well polystyrene were blocked with 300 μL 1% BSA in Milli-Q water for 2 h at 37 °C. After 2 h, wells were rinsed three times with Milli-Q water. Plates were coated immediately prior to use. Clots were prepared using the same method as turbidity analysis, but clots were allowed to form for five times the fibrin generation time to ensure samples were fully clotted. Fibrin clots and surfaces were removed from wells and fixed in 1% paraformaldehyde and 2.5% glutaraldehyde in 0.1 M sodium cacodylate buffer overnight. Samples were then rinsed twice in 0.1 M cacodylate buffer for 15 min each. Samples were dehydrated in a graded series of ethanol (10%, 30%, 50%, 70%, 90%, 95%, and 100%) for 10–15 min each, followed by critical point drying (Electron Microscopy Sciences). Samples were sputter-coated with gold/palladium (Cressington 108A) and imaged on a scanning electron microscope (SEM) (FEI Quanta 600F). ImageJ was used to measure fiber diameter and clot density. Clot density was determined by drawing a line through the image and counting the number of times a fiber crosses the line [36]. The diameter of ten fibers per spot per sample were measured. Three lines of 4 μm in length were drawn per spot per sample. Two samples per sample group were analyzed and the experiment was repeated twice.

### Platelet adhesion and activation

10^8^ platelets/mL in Tyrode’s buffer were incubated on surfaces in a 96-well plate for 2 h at 37 °C. Surfaces were rinsed with Tyrode’s buffer five times to remove nonadherent and loosely bound platelets. The samples were then fixed and prepared for SEM using the same method as for the clots. At least three spots per sample and two samples per sample group were imaged. Blood from two donors was used, and the experiment was repeated twice. ImageJ was used to count the number of adherent platelets and calculate average cell area. The average cell area describes where on average platelets fall on the spectrum of activation. Inactive discoid platelets have a smaller area than active spread platelets, at approximately 2–10 μm^2^ and 20–50 μm^2^, respectively.

## Results and Discussion

The overall goal was to, first, demonstrate that we can produce a universal coating that mimics the pitcher plant SLIPS by using the sticky chemistry provide by mussels (PDA) and, then, to test the biocompatibility of the layer. To assess our coating strategy, we used several complimentary surface analysis techniques to characterize our biomimetic surfaces. The wettability of each surface was probed with water contact angle measurements, while the chemical composition of the layer was determined by XPS. Surface roughness was evaluated using AFM to confirm successful formation of a porous structure. Finally, ordering of specific chemical groups within our PDA SLIPS layer was explored via SFG spectroscopy.

**Figure 2:**
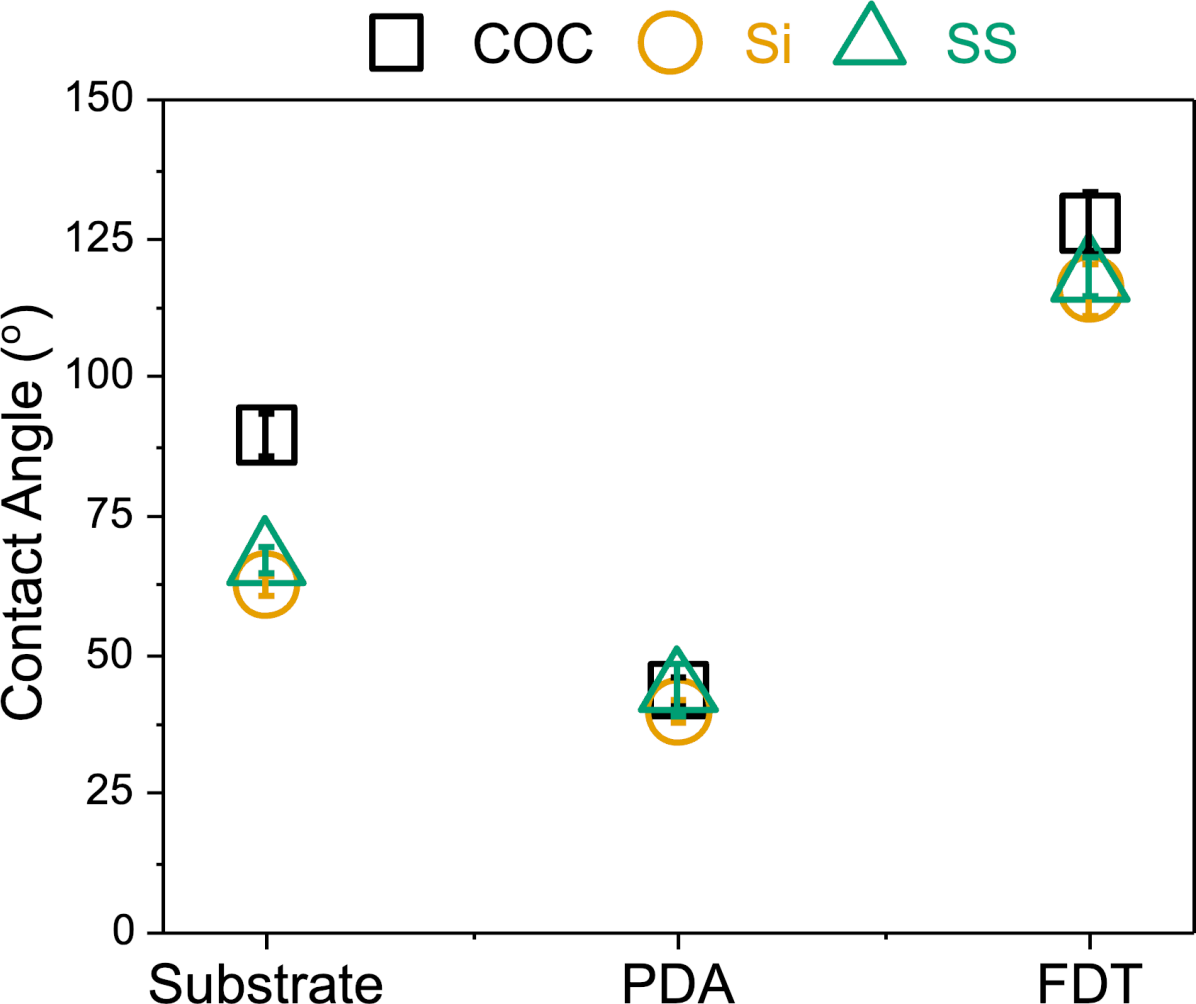
Contact angle measurements during the development of SLIPS coatings. Plot of the static water contact angles for each layer of the coating across the three substrates. Black squares represent COC, yellow circles show Si, and green triangles denote SS. Points are reported as the mean with the bars representative of the standard deviation for a sample size of *n* = 16. The bare substrate, COC (left), is hydrophobic. A layer of polydopamine (PDA) was added to the surface by incubating surfaces overnight with dopamine and Tris. PDA is hydrophilic, so the PDA coating step can be verified via the water contact angle. Fluorinated dodecanethiol (FDT) was conjugated to the PDA surface by incubating overnight. FDT is hydrophobic, so this step can be verified also with water contact angle measurements.

Initially, each functionalization step was assessed through static water contact angle measurements, the results of which can be found in Figure 2. The observed static water contact angles taken from the three different bare substrates varied slightly, with COC as the most hydrophobic one because of its hydrocarbon chemical structure. As these substrates were functionalized with PDA, all three substrates became more wettable. The final functionalization layer (FDT) then coated the surface with electronegative fluorine groups, thereby, creating a more hydrophobic surface [37].

The static water contact angle values collected from the three substrates were (89.3 ± 3.7)° for bare COC, (62.2 ± 1.9)° for Si, and (66.8 ± 2.5)° for SS (Figure 2), all of which were consistent with what was expected [38–41]. The experimental contact angles for substrates following the next functionalization step, the addition of PDA, were (43.3 ± 2.5)° for COC, (39.6 ± 2.1)° for Si, and (43.4 ± 4.7)° for SS, which was also in agreement with other values reported for PDA-coated substrates [19]. The final layer of the coating involved covalently attaching the fluorinated thiol, FDT, to the newly formed PDA film. The reported values for the final layer are (127.6 ± 5.6)° for COC, (115.6 ± 4.5)° for Si, and (117.8 ± 3.5)° for SS.

This dramatic increase in observed hydrophobicity was expected as the fluorine groups in the FDT chains decrease the wettability of the layer. Additionally, a two-tailed *t*-test demonstrated that for each successive functionalization, the contact angles were significantly different from the previous layer (*p* < 0.05). The contact angles from the PDA-coated COC and SS samples were not considered significantly different from one another, and there was neither a statistical difference between the water contact angles observed for the SS and Si samples coated with FDT. However, all substrates coated with the final layer of FDT exhibited water contact angles that exceeded 110°, indicating that we produced of a material with low surface energy suitable for SLIPS on all three substrates [17].

**Figure 3:**
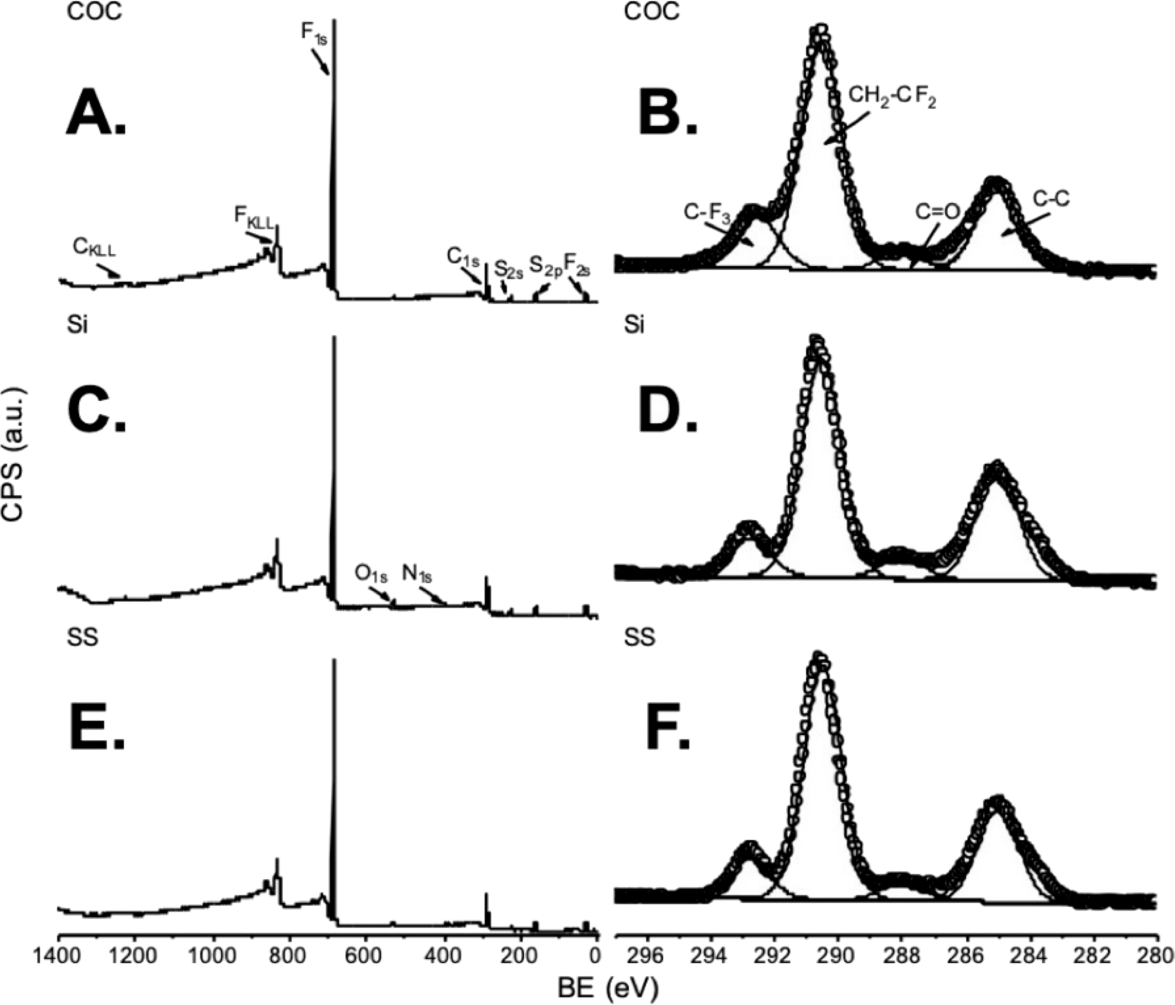
Survey and high-resolution scans of the final layer of the SLIPS coating. (A, C, E) Plots of XPS survey spectra and (B, D, F) high-resolution C 1s scans from the final layer of the coating. The first row (A, B) are the scans for the coating on COC, second row (C, D) on Si, and the final row (E, F) on SS. There are no unexpected peaks in the survey spectrum across all substrates. In the C 1s high-resolution scan, C–C carbon can be found at 285 eV and the fluorinated groups, CF2 and CF3, are observed at 290.6 and 293 eV, respectively. See Supporting Information File 1 for high-resolution S 2p XPS spectra.

Both theoretical and XPS-determined compositions of all three substrates (COC, Si, and SS) functionalized with the PDA–FDT film are reported in Table 1. The atomic compositions for the final layer of the coating were consistent across the three substrates and are similar, thereby, confirming that we were successful with both the PDA and FDT functionalization steps. XPS survey and high-resolution C 1s spectra collected from PDA–FDT-coated substrates can be found in Figure 3. All survey spectra (Figure 3a,c,e) are nearly identical across the three substrates, which suggests a uniform FDT coating across the three different sample types. High-resolution C 1s spectra (Figure 3b,d,f) yield peak envelopes made up of four distinct peaks. These four peaks from high to low binding energies, correspond to C–C/C–H bonds (285 eV), C=O bonds (288 eV), and two peaks related to fluorinated species (CF_2_ at 290.6 eV and CF_3_ at 293 eV) [28,42]. The C–C and C=O peaks likely stem from the PDA layer, while the fluorinated species can be attributed to the FDT layer covalently attached the PDA film. This covalent attachment is also confirmed by the XPS S 2p high-resolution spectra (Supporting Information File 1), which exhibited the same peak envelope across all the three substrates and were representative of a covalent carbon-sulfur bond [43].

**Table 1:** Compositions (atom %) of the final layer of the coating with FDT (*n* = 3) on each of the three different substrates. Theoretical compositions are listed for comparison in the first column.

% Atomic composition				
FDT (*n* = 3)				

element	theoretical	COC	SI	SS

C 1s	37.9	35.2 (2.5)	41.2 (0.5)	38.2 (0.4)
N 1s	0	0 (0.0)	1.4 (0.2)	0.6 (0.1)
S 2p	3.4	5 (1.2)	4.2 (0.2)	4.7 (0.3)
O 1s	0	0.4 (0.2)	2.8 (0.2)	1.5 (0.3)
F 1s	58.6	59.2 (4.0)	50.5 (0.7)	55.1 (0.4)

AFM highlighted the level of roughness at each stage of the coating to show a rougher topography for the final layer. Results for each layer of the coating are shown in Supporting Information File 1, Figure S1 and reported as root mean square roughness (*R*q), a useful metric for comparing surface roughness. The final layer with FDT should increase the surface roughness as only a monolayer is expected to bond. The final value layer of the coating that contains FDT showed *R*q values of 70.5 ± 23.5 nm for COC, 46.9 ± 26 nm for Si, and 47.8 ± 20.7 nm for SS (Supporting Information File 1, Table S1). A two-tailed *t*-test showed that the increase in roughness after each successive coating was significantly different compared to the previous layer, suggesting successful attachment of a porous matrix onto a variety of different substrates. The roughness of all samples with the final layer of the coating were not significantly different from one another, suggesting universal attachment of the SLIPS porous component.

**Figure 4:**
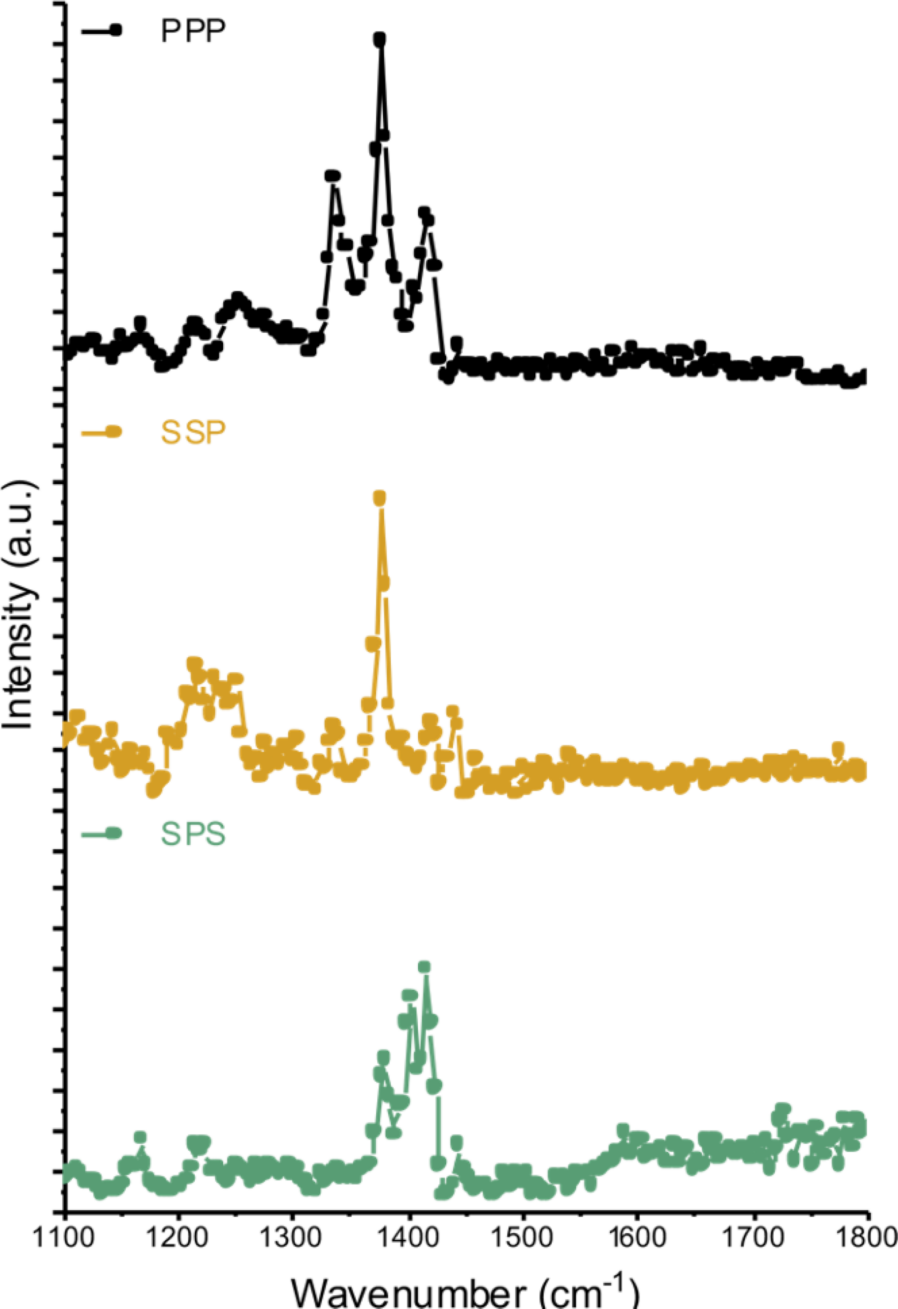
SFG spectra of the final layer in the SLIPs coating. SFG spectra from 1100 to 1800 cm^−1^ from a COC substrate coated with PDA–FDT. The top spectra were collected in ppp, the middle spectra in ssp, and the bottom spectra in sps polarization combinations.

The final structure of oxidative polymerization for dopamine into PDA is not fully agreed upon [20,44–45]. Therefore, information about the presence and ordering of specific chemical groups within the substrate–PDA–FDT layers was provided by SFG spectra collected from the COC–PDA–FDT sample (Figure 4). The stretching region between 1100 and 1850 cm^−1^ contains modes related to specific molecular groups within each layer of the coating. This includes, but is not limited to, C=O carbonyl stretchings from PDA, ring deformations from COC and PDA, and CF_3_ stretchings from FDT. Within the SFG spectra, the carbonyl stretching that stems from the oxidized surface of both COC and PDA was observed at 1730 cm^−1^. Additionally, a ring deformation was observed as a broad peak at around 1600 cm^−1^. This likely originates from ring structures present in both COC and PDA. The feature observed at 1400 cm^−1^ was not readily assigned; however, it was the sole vibrational mode observed for COC in this region and is likely a contribution from order in the bulk. A final mode was assigned to the CF_3_ asymmetric stretching at 1370 cm^−1^ [46].

Observed changes across the SFG spectra collected with different polarization combinations provide information about the orientation of molecular bonds at the interface [26–27]. Here, by collecting SFG spectra from the COC–PDA–FDT sample with ppp, ssp, and sps combinations, we acquired info about the general orientation of both the polymerized dopamine molecules and FDT after being bound to the surface. The CF_3_ asymmetric stretching, specifically in sps, at 1400 cm^−1^ provides evidence that the FDT molecule was oriented out of the interfacial plane. The modes at 1600 cm and 1730 cm^−1^ indicate ring deformation and carbonyl stretching, respectively. The presence of the ring deformation peak at 1600 cm^−1^ in the SPS spectrum suggests that the ring lies in the interfacial plane. This also holds true for the carbonyl stretching at 1730 cm^−1^. Additionally, these modes are not observed in SSP, which further demonstrated that the ring structure and carbonyl groups of PDA lie in the interfacial plane or parallel to the surface. This would support the theory of π–π stacking proposed by Lynge et al., where PDA aggregates form and are joined together through π stacking [45].

As mentioned earlier, quantification of the activation of intrinsic coagulation on SLIPS surfaces have not been fully investigated. For biomaterials, the intrinsic pathway has been shown to activate upon exposure, whereas in vivo the extrinsic pathway dominates [47]. Thus, in this investigation we took one of our newly created PDA SLIPS samples (PDA–FDT–PFD on COC) and characterized it in terms of FXII activation, fibrin generation time, clot morphology, and platelet adhesion and activation. Previously, Badv et al. hypothesized that the prolonged clotting time they observed on a SLIPS-coated catheter was due to reduced activation of the contact system, which implied a lower concentration of FXIIa on their surfaces [18]. Additionally, it has been shown that FXII activation is reduced at hydrophilic surfaces [48], potentially due to trapping of a liquid layer, which reduced protein adhesion. While activation is decreased on hydrophilic surfaces, it is elevated on negatively charged surfaces like glass [49].

**Figure 5:**
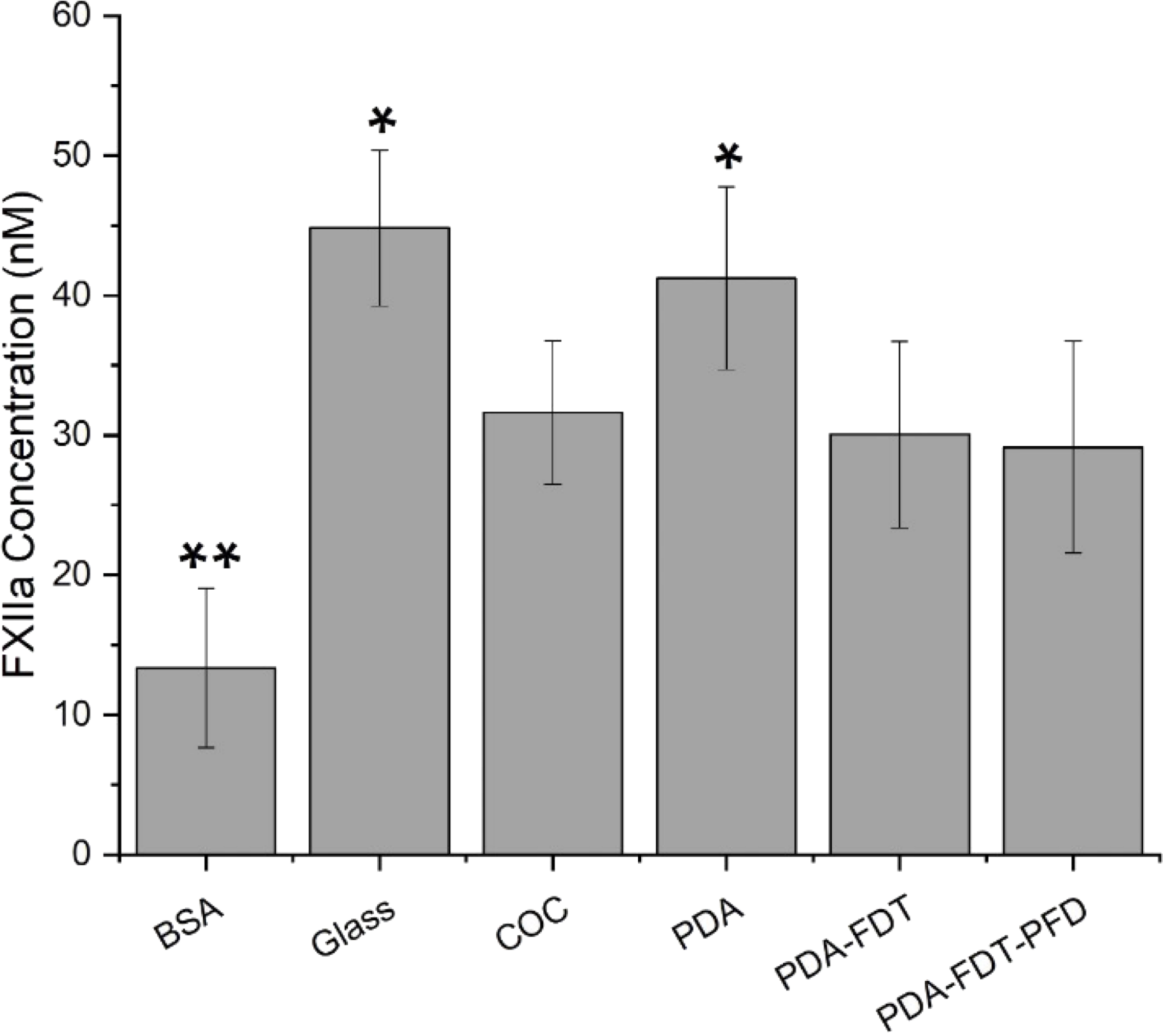
FXIIa concentration after 1 h incubation with HMWK and PK on surfaces. Error bars indicate standard deviation, *n* = 18. COC, PDA–FDT and PDA–FDT–PFD activated 50% less FXII than PDA and glass, but 100% more than BSA. Single asterisk (*) and double asterisk (**) indicate significantly higher or lower activation, respectively (α = 0.05).

FXIIa concentration was determined at each step of our surface modification process. In this work, we used BSA-coated surfaces as a negative control and glass as a positive control. We expected to observe less activation of FXII on PDA SLIPS surfaces compared to untreated surfaces, such as negatively charged glass and COC, because of the prospective omniphobic properties aiding in protein adhesion resistance. FXII did not autoactivate in neat buffer on all PDA SLIPS surfaces but did autoactivate on glass surfaces (Figure 5). A three times increase in FXII activation on bare glass compared to BSA was reported previously, which is consistent with what we observed here [33]. We detected a ≈65% increase in FXII activation on PDA–FDT–PFD compared to BSA and a ≈40% reduction in FXII activation on PDA–FDT–PFD compared to glass. This suggests that there is FXII coming down onto the surface of our coating and activating, though to a lesser degree than on glass. However, we observed no difference in FXII activation between PDA and glass, which is unsurprising given that both surfaces are hydrophilic. There was also no observed difference in FXII activation between COC and PDA–FDT–PFD, suggesting COC exhibits an intrinsic coagulation behavior similar to that of PDA–FDT–PFD.

**Figure 6:**
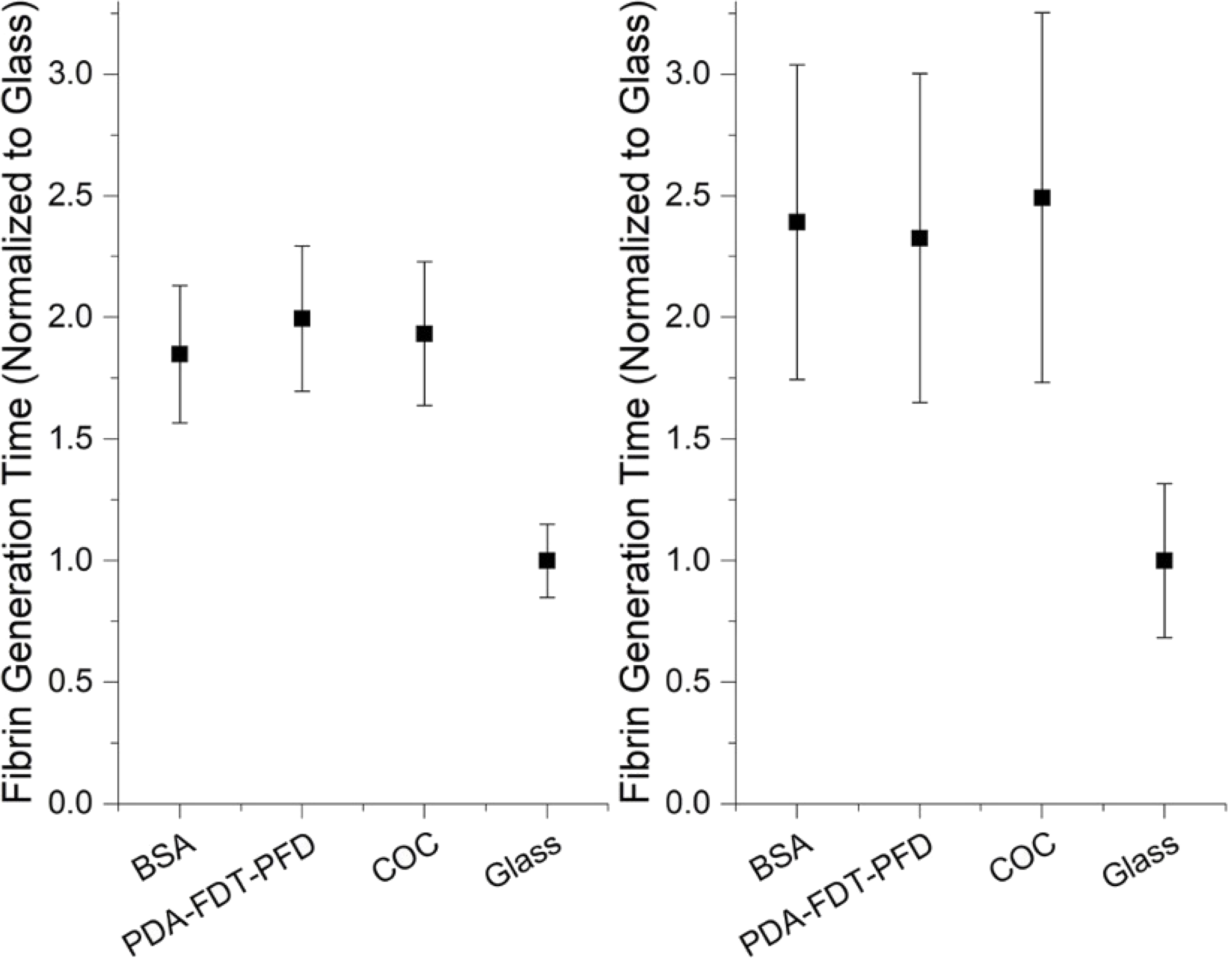
Fibrin generation time on modified surfaces for two donors, A1 (left) and A3 (right). Data was normalized to glass and combined for three runs. Error bars represent standard deviation. Recalcified citrated plasma was incubated with the surfaces at 37 °C. OD was measured every 1 min for 60 min. PPP clotted approximately 2.5 times slower on PDA–FDT–PFD than on glass in all runs, and there was no difference between PDA–FDT–PFD and BSA or COC. Fibrin generation time was defined as the time to reach a 5% increase in OD over the baseline. PDA–FDT–PFD clotted significantly slower than glass for both donors and all runs (α = 0.05).

Next, we tracked the fibrin generation kinetics of PPP across all our experimental and control surfaces (Figure 6). The observed range of fibrin generation time of 5–30 min was consistent with those reported previously using similar methods of turbidimetric quantification on other experimental surfaces [33,50]. We observed that plasma clotted 2.5× slower on PDA–FDT–PFD than glass and there was no significant difference between PDA–FDT–PFD, BSA, or COC. The observed differences in FXII activation between BSA and PDA–FDT–PFD, combined with the lack of observed differences in plasma clotting time, would suggest that PDA–FDT–PFD induces an antithrombogenic pathway that is different than the typical intrinsic coagulation behavior.

**Figure 7:**
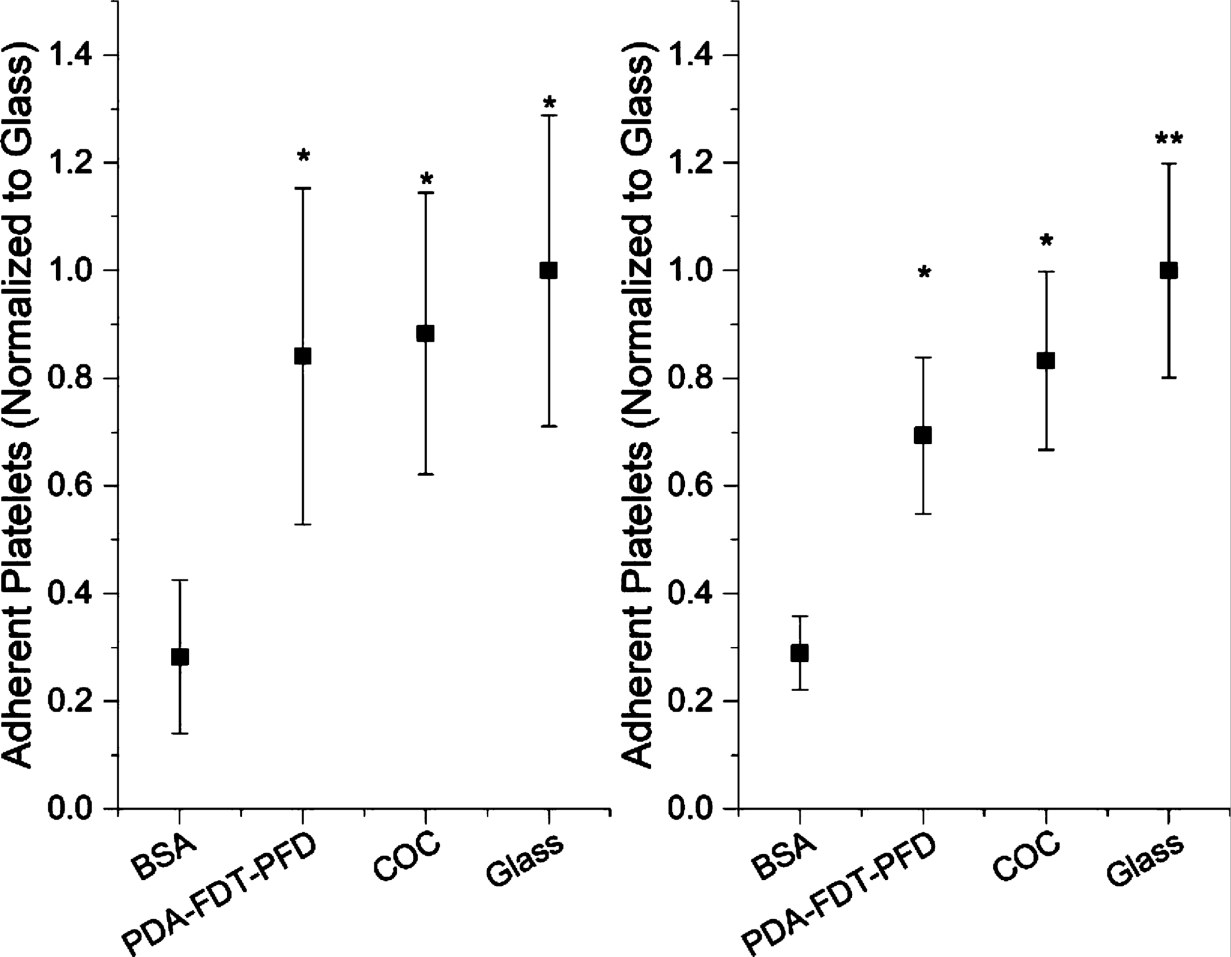
Adherent platelets per 3520 μm^2^ surface area for two donors, A1 (left) and A3 (right). Surfaces were incubated with 10^8^ platelets/mL in Tyrode’s buffer for 90 min. Platelets were counted using SEM. For each donor and each run, two samples per sample group and three spots per sample were imaged. There was approximately 150% lower adhesion to BSA-coated surfaces than to all other surfaces. There was no difference in platelet adhesion between PDA–FDT–PFD, COC, and glass for donor A1, and glass had a significantly higher platelet adhesion than PDA–FDT–PFD and COC for donor A3.

Previously, SLIPS coatings were observed to resist platelet adhesion in whole blood and platelet-rich plasma adhesion assays [4,10]. However, no work has been done previously in a purified platelet system, which allows for identifying the specific interactions between platelets and surfaces independent of protein adsorption or other events that may take place within whole blood and plasma studies. In our purified platelet system, we observed a 150% increase in adherent platelets on PDA–FDT–PFD compared to BSA and a 50% decrease in adherent platelets on PDA–FDT–PFD compared to glass (Figure 7). This suggests that the SLIPS coating is not completely omniphobic as adherent cells are observed.

Fibrin generation time was determined on BSA, glass, bare COC, and PDA–FDT–PFD. We expected to observe a longer fibrin generation time on PDA–FDT–PFD than on glass and COC because of the hypothesized antithrombogenic behavior due to omniphobicity of SLIPS. PPP clotted approximately 2.5 times slower on PDA–FDT–PFD than on glass for all runs, and there was no difference between PDA–FDT–PFD and BSA or COC (Figure 6). Contrastingly, previous studies investigating SLIPS-induced fibrin polymerization found a significant reduction compared to bare surfaces [4]. There was significant variation between donors and between runs, but the trends remained consistent. Differences between runs were not of interest, so data was normalized to the fibrin generation time on glass. Samples were isolated from three distinct draws from each of two donors for a total sample size of 14 per sample per donor.

**Figure 8:**
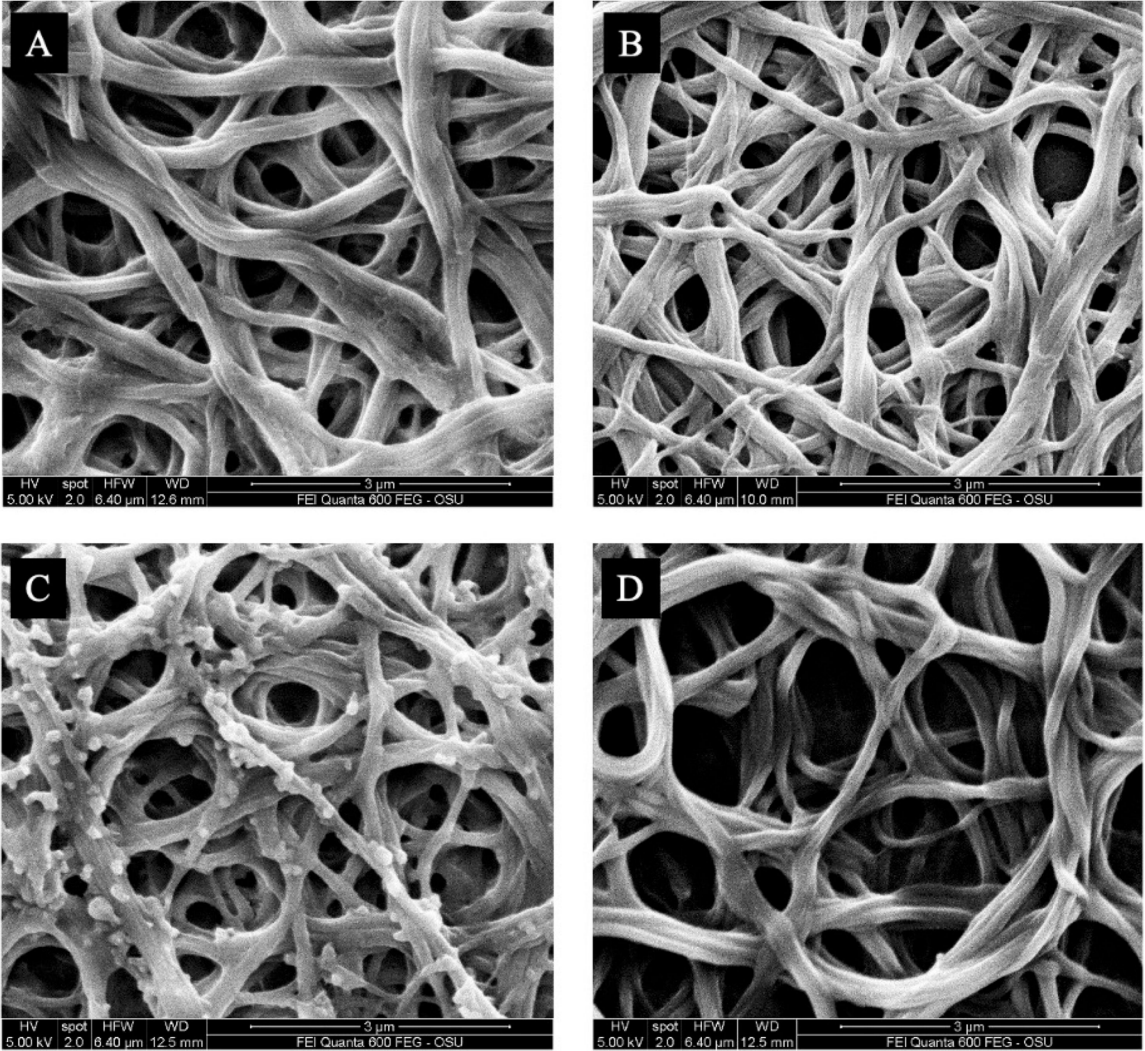
Representative SEM micrographs of clotted plasma on (A) BSA, (B) glass, (C) COC, and (D) PDA–FDT–PFD. Recalcified citrated PPP was incubated with the surfaces for at least ten times the clotting time. All micrographs are from donor A1.

**Figure 9:**
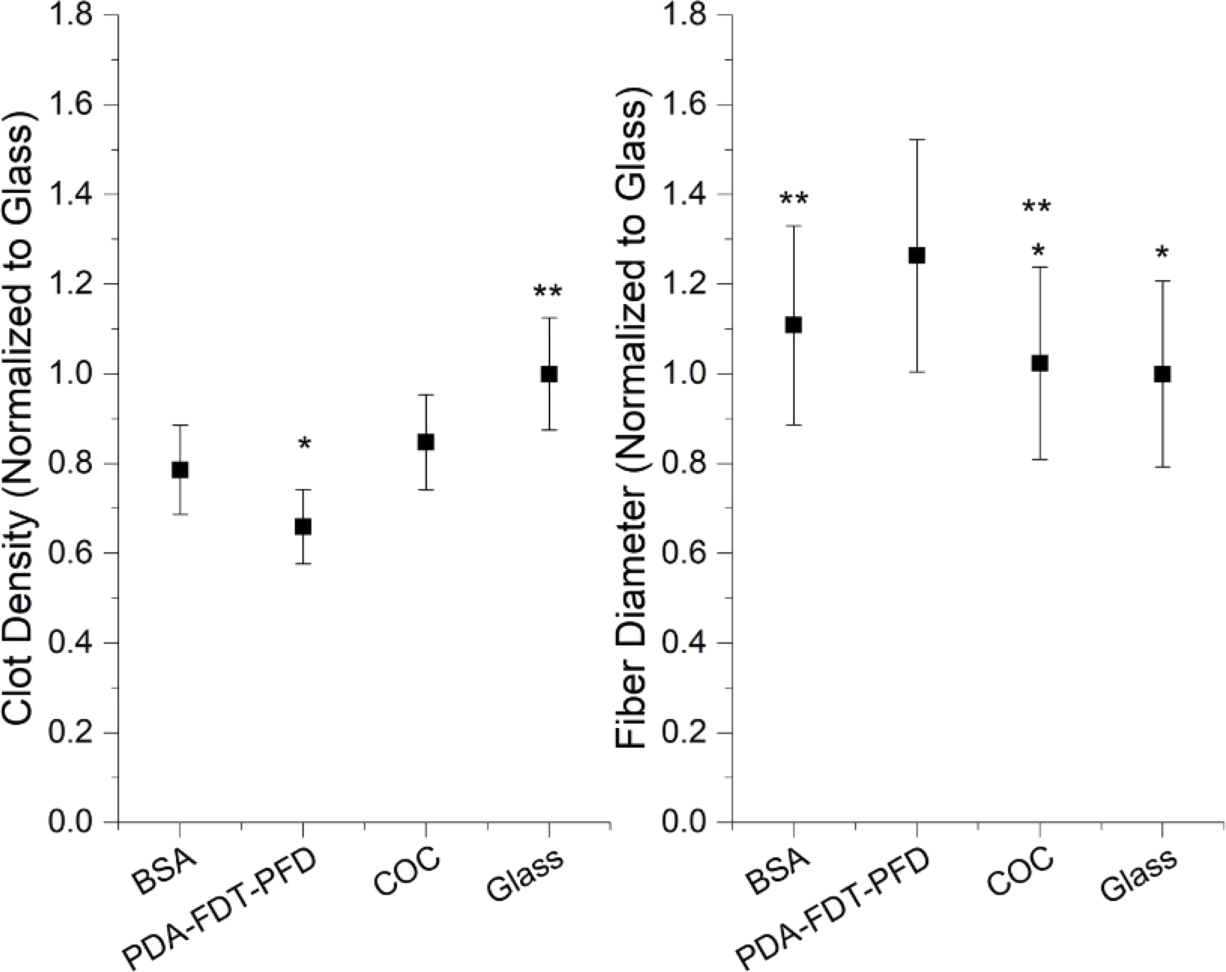
Clot morphology assessed using SEM after incubation of recalcified PPP on surfaces. Data are from a single donor. (Left) Fiber diameter, measured on ten fibers (*n* = 60). (Right) Clot density, defined as the number of times a fiber crosses a line of fixed length. Three lines were drawn per spot (*n* = 18). PDA–FDT–PFD yielded significantly higher fiber diameter and lower clot density than glass, COC and BSA (α = 0.05).

Clot fiber diameter and crosslinking density measurements were taken from SEM micrographs. Representative SEM micrographs are shown in Figure 8. Less stable clots exhibit larger fiber diameters and more porous fibrin networks. Therefore, PDA–FDT–PFD was expected to produce clots with larger fiber diameters and less crosslinked networks compared to the bare glass and COC surfaces. Data was normalized to glass, and PDA–FDT–PFD yielded an approximately 20% higher fiber diameter and 25% lower clot density than glass (Figure 9). PDA–FDT–PFD also led to significantly higher fiber diameter and lower clot density than BSA and COC, suggesting that clots formed on PDA–FDT–PFD are less stable and easier to break down than clots formed on glass, COC, and BSA [36]. Similar surface-induced thrombus properties were observed in other SLIPS applications, where, in comparison to untreated surfaces, SLIPS-treated surfaces under dynamic blood flow yielded significantly lower thrombi weights [4].

**Figure 10:**
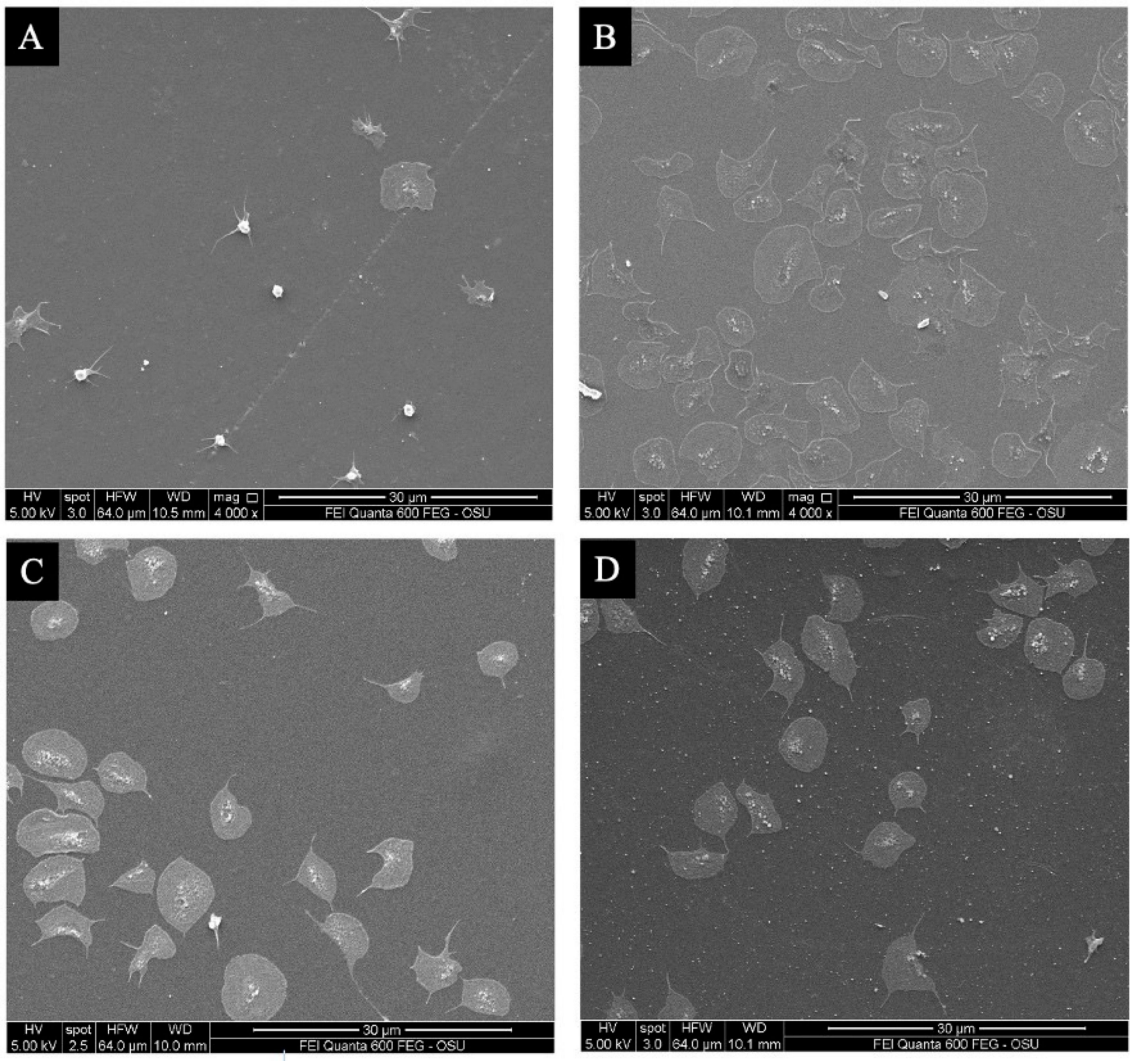
Representative SEM micrographs of adherent platelets on (A) BSA, (B) glass, (C) COC, and (D) PDA–FDT–PFD. Surfaces were incubated with 10^8^ platelets/mL in Tyrode’s buffer for 90 min.

Finally, platelet adhesion was quantified from SEM micrographs. As mentioned earlier, SLIPS surfaces have been shown to resist platelet adhesion because of their omniphobic properties [4,10]. Therefore, we expected to observe little to no adherent platelets on the PDA–FDT–PFD surfaces. To test this, purified platelets were incubated with surfaces for 90 min, rinsed three times, fixed, and dehydrated in preparation for imaging. The horizontal field width was set to 64 μm, the surface area for analysis was 3520 μm^2^ (Figure 10), and data was normalized to glass. There was approximately 150% lower adhesion to BSA-coated surfaces than to all other surfaces. There was no difference in platelet adhesion between PDA–FDT–PFD, COC, and glass for donor A1. However, glass exhibited significantly higher platelet adhesion than PDA–FDT–PFD and COC for donor A3 (Figure 7).

Combined, these biocompatibility assays demonstrate that this formulation of a SLIPS coating is not completely omniphobic as FXII activated on the surface and platelets adhered to the coating. The observed prolonged fibrin generation time in PPP, however, suggests that SLIPS could still exhibit antithrombogenic behavior. The FXII assay and platelet adhesion studies were performed in a purified system to gather information on isolated surface interactions, whereas the fibrin generation study was performed using PPP to investigate comprehensive hematologic behavior of the surfaces. This suggests that the SLIPS coating could have a high affinity for a passivating blood protein or low affinity for an active procoagulant. More work on specific coagulation factor adsorption and activation, such as thrombin, fibrinogen, and complement system proteins is needed to fully characterize the surface. Studies on FXII and platelet adhesion in plasma and whole blood models would provide more insight into observed activation in a purified system.

## Supporting Information

File 1 Additional figures and table.

## Data Availability

The data that supports the findings of this study is available from the corresponding author upon reasonable request.
