# Supplementary material for: A biomimetic approach towards a universal slippery liquid infused surface coating
Source: Beilstein J Nanotechnol. 2024 Nov 8;15:1376–89. doi: 10.3762/bjnano.15.111 (PMC11552445; doi:10.3762/bjnano.15.111)
Supplement: File 1 — Additional figures and table. [file Beilstein_J_Nanotechnol-15-1376-s001.pdf]

BEILSTEIN JOURNAL OF NANOTECHNOLOGY

Supporting Information

for

A biomimetic approach towards a universal slippery liquid infused surface coating

Ryan A. Faase, Madeleine H. Hummel, AnneMarie V. Hasbrook, Andrew P. Carpenter and Joe E. Baio

Beilstein J. Nanotechnol. **2024,** *15,* 1376–1389. doi:10.3762/bjnano.15.111

Additional figures and table

License and Terms: This is a supporting information file under the terms of the Creative Commons Attribution License (https://creativecommons.org/licenses/by/4.0). Please note that the reuse, redistribution and reproduction in particular requires that the author(s) and source are credited and that individual graphics may be subject to special legal provisions.
The license is subject to the *Beilstein Journal of Nanotechnology* terms and conditions: (https://www.beilstein-journals.org/bjnano/terms)

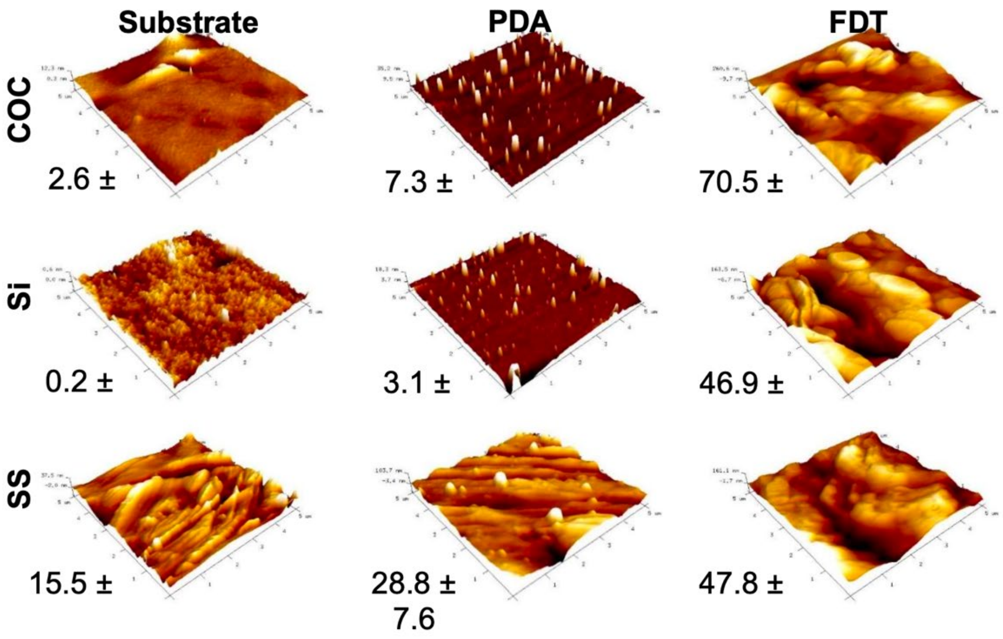

Figure S1: Image array of surface roughness for SLIPS coating. AFM scans for each layer of the coating from each of the three substrates. Substrates are listed in each column where the first row represents scans for each substrate, the second column represents PDA, and the final column is of the scans with FDT. Values are reported as means ± standard deviation.

Table S1: SLIPS coating AFM results. Table of results with root mean square roughness (R_q) values for each 5 × 5 µm scan for every sample. Averages and standard deviations are reported with an *n* = 6 for the bare substrate and PDA, where there was an *n* = 12 for the layer with FDT.

**R_q (nm)**	**COC**	**Si**	**SS**
Substrate (*n* = 6)	2.6 (1.1)	0.2 (0.0)	15.5 (7.4)
PDA (*n* = 6)	7.3 (3.1)	3.1 (0.5)	28.8 (7.6)
FDT (*n* = 12)	70.5 (23.5)	46.9 (26.0)	47.8 (20.7)

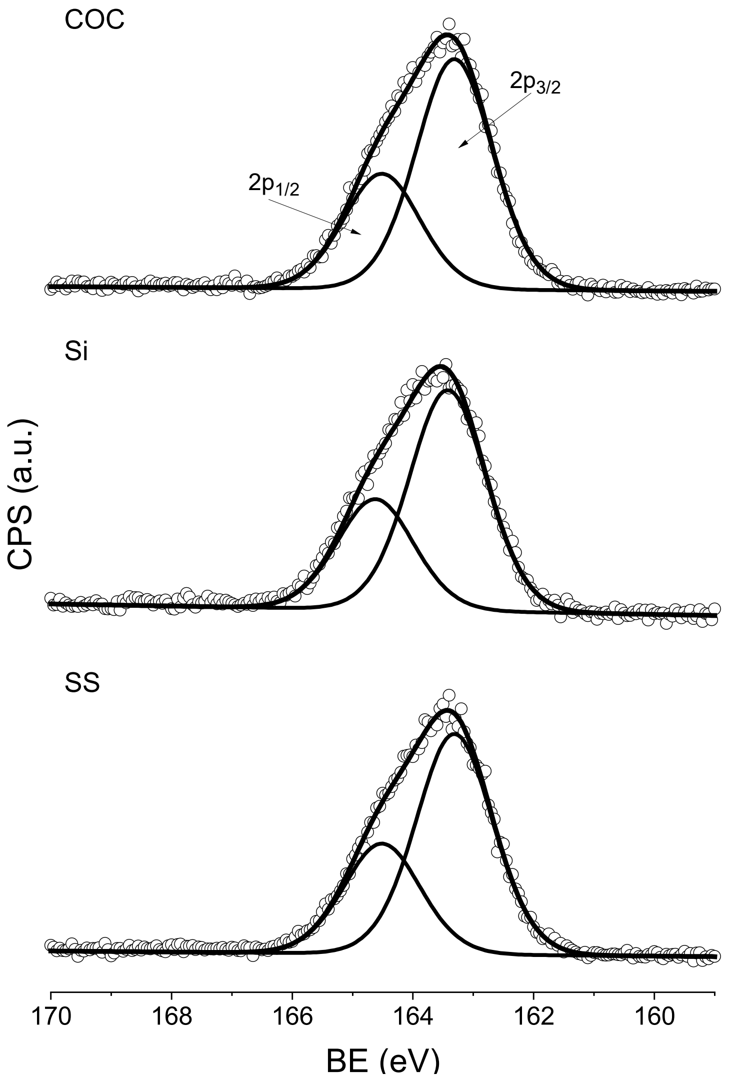

Figure S2: High-resolution spectra of the S 2p region for the final layer of the coating. The top spectrum represents the coating on COC, the middle spectrum on Si, and the bottom spectrum on SS. The $2p_{3/2}$ component at 163.5 eV and the $2p_{1/2}$ component at 164.7 eV are indicative of a covalent bond between carbon and sulfur.
